# Being More Realistic about the Public Health Impact of Genomic Medicine

**DOI:** 10.1371/journal.pmed.1000347

**Published:** 2010-10-12

**Authors:** Wayne D. Hall, Rebecca Mathews, Katherine I. Morley

**Affiliations:** 1 University of Queensland Centre for Clinical Research, The University of Queensland, Herston, Queensland, Australia; 2 Queensland Brain Institute, The University of Queensland, St. Lucia, Queensland, Australia; 3 Wellcome Trust Sanger Institute, Wellcome Trust Genome Campus, Hinxton, Cambridge, United Kingdom; 4 Centre for Molecular, Environmental, Genetic and Analytic Epidemiology, School of Population Health, The University of Melbourne, Victoria, Australia

## Abstract

Wayne Hall and colleagues discuss the limitations of genomic risk prediction for population-level preventive health care.

**Summary Points**

Before genomic information is used in public health screening, it must be shown that:

- such information predicts disease risk better than phenotypic information;
- cost-effective interventions exist for those at increased genetic risk;
- these interventions are more cost-effective than population-level interventions;
- genetic risk information motivates desired behaviour change.

Currently there are no examples of genetic screening for disease risk that satisfy these criteria.

In the 1990s, during the era of the Human Genome Project, many researchers were very optimistic about the capacity of such large-scale genetic projects to revolutionize the prevention of disease (e.g., [1],[2]). Many predicted that whole populations would be screened for their genetic susceptibility to common diseases, such as cancer and heart disease. Healthy individuals who carried susceptibility alleles would be advised to change their behaviour (e.g., exercise more, maintain a healthier diet, stop smoking), or be given drugs or other treatments to reduce their risks of developing these diseases.

Ten years on, genome-wide association studies (GWAS) have changed our understanding of the aetiology of many common diseases such as type 1 diabetes and obesity, but they have not identified major susceptibility alleles for most common diseases. With a few exceptions, susceptibility alleles for the most common human diseases have proven to be very weakly predictive of disease risk, with odds ratios for individual alleles typically ranging from 1.1 to 1.6 [3]–[5]. The susceptibility “genes” identified to date account for only a small percentage of the known genetic variation in disease risk, and the relationship between these genetic variants and environmental risk factors has yet to be fully investigated [3],[6].

Despite these limitations, many researchers continue to advocate the use of genetic information to predict disease risk (e.g., [7]) and a number of private companies now offer this as a service on an individual basis. Is genetic risk prediction feasible from a public health perspective, in the way that many originally envisaged?

In principle, individual genetic variants could potentially provide reasonable prediction of disease risk, if the findings for multiple susceptibility alleles were combined statistically [8]–[12]. Modelling of this approach has produced conflicting assessments about its likely utility (e.g., [13]–[16]). The same has been true of empirical tests of genetic prediction of common complex traits (see [17] for a list of recent studies).

In many contexts, information from multiple genetic variants does not appear to provide better prediction than known risk factors such as family history or environmental risks. For instance, Lango and colleagues [18] found that 18 genetic variants that individually predicted an increased risk of diabetes were not able to discriminate between type 2 diabetes cases and controls and only marginally improved upon predictions using age, body mass index (BMI), and sex. Studies of genomic prediction of coronary heart disease and cardiovascular events have also found that genotypic information is less predictive of disease risk than are age, blood pressure, cholesterol, triglycerides, cigarette use, and diabetes [19],[20]. On the other hand, there are some cases in which genotypic information discriminates between subpopulations that differ markedly in disease risk. For instance, Pharoah and colleagues [21] found that combinations of breast cancer susceptibility alleles discriminated between women at low and high risk of breast cancer on the basis of family history.

The difference in success of prediction between these empirical studies is perhaps not surprising; disease prevalence and heritability are important determinants of the clinical utility of a genetic test. Also, genetic associations often vary by population, because population level variations in location, ethnicity, age, and other factors influence the prevalence not only of genetic risk factors for common diseases, but also of environmental and behavioural risk factors for common diseases, such as tobacco and alcohol use, diet, and exercise [22]. Consequently, the predictive capacity and clinical utility of genetic tests will depend upon the population in which they are used and the disease(s) for which risk is being predicted. Thus it is not possible to make any overarching statement about the utility of predictive genetic tests. We can, however, outline some of the likely constraints on the implementation of population-wide screening using genetic tests to predict disease risk.

## Constraints on Public Health Impact

### Cost-Effectiveness of Predictive Genomic Medicine

Advocates of the preventive use of genetic risk information often simply assume that preventive interventions will be cost-effective if genotypic information can be shown to predict disease risk (e.g. [7]). From a public health perspective, however, population-wide screening (genetic or otherwise) is ethically justifiable only if there is an efficacious and cost-effective intervention to prevent the disorder in those who are identified as being at increased risk [23]–[25]. For common cancers, such as colorectal and breast cancer, regular monitoring and early treatment can reduce mortality, and there are also preventive medications for hypercholesterolemia and high blood pressure.

However, even if efficacious interventions are available, we need large controlled trials to assess whether providing these interventions to asymptomatic individuals at increased genetic risk is more cost-effective than treating all persons displaying physiological risk factors (such as elevated blood pressure or cholesterol) [26],[27]. Prostate-specific antigen (PSA) screening provides a cautionary example. A positive PSA test is modestly predictive of the risk of developing invasive cancer of the prostate [28] but epidemiological modelling shows that 1,500 men need to be screened to prevent one death from prostate cancer and this death would be averted at the cost of unnecessary surgery for 80 low-risk men whose quality of life would be seriously impaired [29].

### Behavioural Impacts of Genetic Risk Information

Some advocates of genomic medicine simply assume that giving genetic risk information will prompt individuals to change their behaviour in desired directions [26],[30]. It is not clear that this is the case [8],[31]. Information about genetic susceptibility to disease only seems to have, at most, a small negative psychological impact on result recipients ([32]–[34]), but inappropriate communication of genetic risk information may actually undermine individuals' beliefs about their ability to change their behaviour [35],[36].

There is some evidence that genetic risk information may make individuals assume that prevention requires pharmacological intervention [37]. For example, genetic risk information about familial hypercholesterolaemia [35] increased individuals' beliefs that the best way to reduce their risk was to use lipid-lowering medication rather than to change their diet or increase exercise. Wright et al. [36] found that smokers who were told that they had a genetic predisposition to nicotine dependence were more likely to believe that they needed to use a drug to quit smoking, despite the fact that most smokers quit unaided [38].

It is also unclear whether genetic risk information produces sustained changes in risk behaviour [34]. Studies have shown that testing positively for a genetic predisposition to lung cancer increased smoking cessation attempts [39] and reduced the number of cigarettes smoked [40] but neither of these changes lasted for more than six months. Another study reported genetic testing for hereditary breast and ovarian cancer promoted healthy lifestyle changes in approximately half of all patients tested, but behaviour change did not differ between carriers and non-carriers of the gene [41].

### Competing Population Health Strategies

Predictive genomic medicine adopts a “high risk” strategy [25] that targets interventions at individuals who are at the highest risk of developing a disease [9]. Public health professionals are concerned that an uncritical embrace of “high risk” strategies will displace more effective strategies that aim to shift population distributions of risk exposures, for example by reducing the population prevalence of cigarette smoking, per capita alcohol consumption, average blood pressure, or the consumption of energy-dense foods [25].

Population-based tobacco control strategies, such as taxing cigarettes and reducing opportunities to smoke, have halved cigarette smoking rates in Australia [42] and the US [43] over the past three decades. These population-based strategies are more efficient than high-risk strategies [25] because fewer resources are needed to increase taxes on tobacco products, ban cigarette advertising, and restrict opportunities to smoke than are needed to screen whole populations in order to identify and intervene with the minority at high genetic risk of becoming nicotine dependent or developing tobacco-related diseases, if they smoke tobacco [9],[44].

There are similar arguments for the greater efficiency of population-based strategies in reducing risky alcohol use, obesity, and diabetes. These strategies aim to reduce population access to cheap energy-dense foods and increase opportunities to exercise. Based on the successful experiences in tobacco control, such strategies will probably include: increased taxes on, reductions in the promotion of, and decreased availability of, energy-dense foods; and redesigning urban environments to reduce sedentary behaviour and increase opportunities for incidental exercise in everyday life [45],[46].

### Subversive Uses of Genomic Risk Information

Public health professionals are also concerned about the potential misuse of genetic risk information by industries that wish to promote harmful forms of consumption (tobacco, alcohol, energy dense foods, and gambling). These industries are likely to advocate for population-wide genetic screening in order to undermine public health policies that will reduce the use of their products in the population [47].

Analyses of industry documents [48] demonstrate that this is why the tobacco industry funded behavioural and molecular genetic research on smoking and tobacco-related disease in the 1970s and 1980s. A strategic decision was taken to promote genetic explanations of tobacco-related disease (initially promoted by the geneticist R.A. Fisher). By locating the risks of smoking in the genome of the individual smokers, this research could be used to exonerate tobacco smoking as a cause of disease [48].

The alcohol industry has also promoted the idea that alcohol-related problems only occur in a minority of genetically vulnerable drinkers [49]. The policy implication favoured by the industry is that alcohol problems are better addressed by identifying and intervening with problem drinkers rather than adopting effective strategies for reducing population-level alcohol consumption, such as increased taxation and reduced availability of alcohol [50]. The gambling industry has recently funded research into the genetics and neurobiology of problem gambling [51] for presumably similar strategic reasons. The food industries will find genetic explanations of obesity similarly useful in undermining population-wide strategies to reduce obesity by modifying obesogenic environments [46].

### The Necessity for Technology Evaluation

The major public health challenge in formulating a policy toward population-wide genomic screening will be in discovering how to obtain whatever public health benefits genomic medicine delivers for common diseases without undermining effective population health policies that reduce exposure to the common risk factors responsible for the high prevalence of these diseases in developed countries [9].

Public health utility should, however, be differentiated from the usefulness of genetic information in a medical context. Research may identify low-frequency genetic variants with large effects that can be used in matching treatments to patients in clinical settings. Rare variants may also identify promising new targets for drugs to treat common diseases. But the usefulness of such variants for public health screening will be limited because of their low frequency in the population.

Although early research has not shown a strong improvement in predictive power when genetic and environmental factors are combined to estimate disease risk, the use of Mendelian randomisation may enable epidemiologists to assess the causal role of environmental factors in common diseases [52]. If the relationship between genetic and environmental risk factors can be better characterised, the utility of public health screening tests may be improved by combining phenotypic and environmental information and administering such tests to subsets of the population who have other indicators of increased risk (such as a history of early-onset disease in first-degree relatives).

But it is clear that genetic screening of whole populations is unlikely to transform preventive health in the ways predicted 10 years ago. The integration of individual genomic risk prediction into public health disease prevention strategies will require good evidence that this approach improves on the cost-effectiveness of existing population level interventions. The utility and cost-effectiveness of predictive genomics, like any other new health technology, should be evaluated disease-by-disease and population-by-population. Its utility will depend not only upon the costs of genetic screening (which have fallen rapidly) but also on: the effectiveness of treating those at increased risk; the morbidity and mortality that these preventive interventions avert and cause; and on our ability to prevent the subversive use of genomic information by interested industries to undermine effective public health policies. Until we have a much stronger evidence base, and more data on interactions between genotypes and common environmental exposures, advocates of genomic medicine should be much more modest than some have been in the claims they make about its likely impacts upon population health.

## Abbreviations

BMI: body mass index
GWAS: genome-wide association studies
PSA: prostate-specific antigen

## References

[1] Collins FS (1999). Shattuck lecture—medical and societal consequences of the Human Genome Project.. N Engl J Med.

[2] van Ommen GJ, Bakker E, den Dunnen JT (1999). The human genome project and the future of diagnostics, treatment, and prevention.. Lancet.

[3] Donnelly P (2008). Progress and challenges in genome-wide association studies in humans.. Nature.

[4] Hindorff LA, Sethupathy P, Junkins HA, Ramos EM, Mehta JP (2009). Potential etiologic and functional implications of genome-wide association loci for human diseases and traits.. Proc Natl Acad Sci U S A.

[5] Manolio TA, Brooks LD, Collins FS (2008). A HapMap harvest of insights into the genetics of common disease.. J Clin Invest.

[6] Manolio TA, Collins FS, Cox NJ, Goldstein DB, Hindorff LA (2009). Finding the missing heritability of complex diseases.. Nature.

[7] Gulcher J, Stefansson K (2010). Genetic risk information for common diseases may indeed be already useful for prevention and early detection.. Eur J Clin Invest.

[8] Khoury MJ (2003). Genetics and genomics in practice: the continuum from genetic disease to genetic information in health and disease.. Genet Med.

[9] Khoury MJ, Yang Q, Gwinn M, Little J, Flanders WD (2004). An epidemiological assessment of genomic profiling for measuring susceptibility to common diseases and targeting interventions.. Genet Med.

[10] Kraft P, Hunter DJ (2009). Genetic risk prediction—are we there yet?. N Engl J Med.

[11] Pharoah PD, Antoniou A, Bobrow M, Zimmern RL, Easton DF (2002). Polygenic susceptibility to breast cancer and implications for prevention.. Nat Genet.

[12] Visscher PM (2008). Sizing up human height variation.. Nat Genet.

[13] Clayton DG (2009). Prediction and interaction in complex disease genetics: experience in type 1 diabetes.. PLoS Genet.

[14] Janssens AC, Aulchenko YS, Elefante S, Borsboom GJ, Steyerberg EW (2006). Predictive testing for complex diseases using multiple genes: fact or fiction?. Genet Med.

[15] Wray NR, Goddard ME, Visscher PM (2007). Prediction of individual genetic risk to disease from genome-wide association studies.. Genome Res.

[16] Wray NR, Yang J, Goddard ME, Visscher PM (2010). The genetic interpretation of area under the ROC curve in genomic profiling.. PLoS Genet.

[17] Janssens AC, van Duijn CM (2009). Genome-based prediction of common diseases: methodological considerations for future research.. Genome Med.

[18] Lango H, Palmer CN, Morris AD, Zeggini E, Hattersley AT (2008). Assessing the combined impact of 18 common genetic variants of modest effect sizes on type 2 diabetes risk.. Diabetes.

[19] Kathiresan S, Melander O, Anevski D, Guiducci C, Burtt NP (2008). Polymorphisms associated with cholesterol and risk of cardiovascular events.. N Engl J Med.

[20] Van der Net JB, Janssens ACJW, Sijbrands EJG, Steyerberg EW (2009). Value of genetic profiling for the prediction of coronary heart disease.. Am Heart J.

[21] Pharoah PD, Antoniou AC, Easton DF, Ponder BA (2008). Polygenes, risk prediction, and targeted prevention of breast cancer.. N Engl J Med.

[22] Visscher PM, Hill WG, Wray NR (2008). Heritability in the genomics era—concepts and misconceptions.. Nat Rev Genet.

[23] Evans JP, Skrzynia C, Burke W (2001). The complexities of predictive genetic testing.. BMJ.

[24] Khoury MJ, McCabe LL, McCabe ER (2003). Population screening in the age of genomic medicine.. N Engl J Med.

[25] Rose G (1992). The strategy of preventive medicine.

[26] Haga SB, Khoury MJ, Burke W (2003). Genomic profiling to promote a healthy lifestyle: not ready for prime time.. Nat Genet.

[27] Ransohoff DF, Khoury MJ (2010). Personal genomics: information can be harmful.. Eur J Clin Invest.

[28] Fiorentino M, Capizzi E, Loda M (2010). Blood and tissue biomarkers in prostate cancer: state of the art.. Urol Clin North Am.

[29] Barratt AL, Stockler MR (2009). Screening for prostate cancer: explaining new trial results and their implications to patients.. Med J Aust.

[30] Hunter DJ, Khoury MJ, Drazen JM (2008). Letting the genome out of the bottle—will we get our wish?. N Engl J Med.

[31] Peto J (2001). Cancer epidemiology in the last century and the next decade.. Nature.

[32] Green RC, Roberts JS, Cupples LA, Relkin NR, Whitehouse PJ (2009). Disclosure of APOE genotype for risk of Alzheimer's disease.. N Engl J Med.

[33] Cameron LD, Sherman KA, Marteau TM, Brown PM (2009). Impact of genetic risk information and type of disease on perceived risk, anticipated affect, and expected consequences of genetic tests.. Health Psychol.

[34] McBride CM, Bowen D, Brody LC, Condit CM, Croyle RT (2010). Future health applications of genomics: priorities for communication, behavioral, and social sciences research.. Am J Prev Med.

[35] Senior V, Marteau TM, Weinman J (2000). Impact of genetic testing on causal models of heart disease and arthritis: an analogue study.. Psychol Health.

[36] Wright AJ, Weinman J, Marteau TM (2003). The impact of learning of a genetic predisposition to nicotine dependence: an analogue study.. Tob Control.

[37] Marteau TM, Weinman J (2006). Self-regulation and the behavioural response to DNA risk information: a theoretical analysis and framework for future research.. Soc Sci Med.

[38] Chapman S, MacKenzie R (2010). The global research neglect of unassisted smoking cessation: causes and consequences.. PLoS Med.

[39] McBride CM, Bepler G, Lipkus IM, Lyna P, Samsa G (2002). Incorporating genetic susceptibility feedback into a smoking cessation program for African-American smokers with low income.. Cancer Epidemiol Biomarkers Prev.

[40] Sanderson SC, Humphries SE, Hubbart C (2008). Psychological and behavioural impact of genetic testing smokers for lung cancer risk: a phase II exploratory trial.. J Health Psychol.

[41] Watson M, Foster C, Eeles R, Eccles D, Ashley S (2004). Psychosocial impact of breast/ovarian (BRCA1/2) cancer-predictive genetic testing in a UK multi-centre clinical cohort.. Br J Cancer.

[42] White V, Hill D, Siahpush M, Bobevski I (2003). How has the prevalence of cigarette smoking changed among Australian adults? Trends in smoking prevalence between 1980 and 2001.. Tob Control.

[43] Pierce JP, Gilpin EA, Emery SL, White MM, Rosbrook B (1998). Has the California tobacco control program reduced smoking?. JAMA.

[44] Hall WD, Madden P, Lynskey M (2002). The genetics of tobacco use: methods, findings and policy implications.. Tob Control.

[45] Chaloupka FJ (2010). Beyond tax: the need for research on alcohol pricing policies.. Addiction.

[46] Krueger H, Williams D, Kaminsky B, McLean D (2007). The health impact of smoking and obesity and what to do about it.

[47] Hall WD, Gartner CE, Carter A (2008). The genetics of nicotine addiction liability: ethical and social policy implications.. Addiction.

[48] Gundle KR, Dingel MJ, Koenig BA (2010). ‘To prove this is the industry's best hope’: big tobacco's support of research on the genetics of nicotine addiction.. Addiction.

[49] Hall WD (2005). British drinking: a suitable case for treatment?. BMJ.

[50] Babor T, Miller P, Edwards G (2010). Vested interests, addiction research and public policy.. Addiction.

[51] Vrecko S (2008). Capital ventures into biology: biosocial dynamics in the industry and science of gambling.. Econ Soc.

[52] Davey Smith G, Ebrahim S, Lewis S, Hansell AL, Palmer LJ (2005). Genetic epidemiology and public health: hope, hype, and future prospects.. Lancet.

